# Characteristics of healthy Japanese young adults with respect to recognition of facial expressions: a preliminary study

**DOI:** 10.1186/s40359-023-01281-5

**Published:** 2023-08-17

**Authors:** Tomoko Hama, Michihiko Koeda

**Affiliations:** 1 Department of Medical Technology, Ehime Prefectural University of Health Sciences, 543 Takoda, Tobe-Cho, Iyo-Gun, Ehime, 791-2101 Japan; 2 Department of Neuropsychiatry, Graduate School of Medicine, Nippon Medical School, 1-1-5, Sendagi, Bunkyo-Ku, Tokyo, 113-8603 Japan; 3 Department of Neuropsychiatry, Nippon Medical School Tama Nagayama Hospital, 1-7-1, Nagayama, Tama, Tokyo, 206-8512 Japan

**Keywords:** Facial expression recognition, Emotion, FACS, Japanese, Culture

## Abstract

**Background:**

Emotional cognitive impairment is a core phenotype of the clinical symptoms of psychiatric disorders. The ability to measure emotional cognition is useful for assessing neurodegenerative conditions and treatment responses. However, certain factors such as culture, gender, and generation influence emotional recognition, and these differences require examination. We investigated the characteristics of healthy young Japanese adults with respect to facial expression recognition.

**Methods:**

We generated 17 models of facial expressions for each of the six basic emotions (happiness, sadness, anger, fear, disgust, and surprise) at three levels of emotional intensity using the Facial Acting Coding System (FACS). Thirty healthy Japanese young adults evaluated the type of emotion and emotional intensity the models represented to them.

**Results:**

Assessment accuracy for all emotions, except fear, exceeded 60% in approximately half of the videos. Most facial expressions of fear were rarely accurately recognized. Gender differences were observed with respect to both faces and participants, indicating that expressions on female faces were more recognizable than those on male faces, and female participants had more accurate perceptions of facial emotions than males.

**Conclusion:**

The videos used may constitute a dataset, with the possible exception of those that represent fear. The subject’s ability to recognize the type and intensity of emotions was affected by the gender of the portrayed face and the evaluator’s gender. These gender differences must be considered when developing a scale of facial expression recognition.

## Backgrounds

Affective cognitive impairment is a core clinical symptom of many neurological and psychiatric disorders [1–3]. Emotion is expressed through the face and voice. The inability to recognize facial expressions of emotions is closely associated with neurological and psychiatric disorders [4–8]. Therefore, the assessment of facial emotion recognition is useful for clarifying disease pathogenesis. Currently, mental health care is based on a disease-specific approach. However, many mental illnesses, such as depression, schizophrenia, and other affective disorders, and psychiatric symptoms, such as low motivation, depression, and cognitive impairment, share extensive commonalities [9–11]. Disparate diagnostic methods developed for various diseases are inadequate for such patients and treatment should focus on specific clinical conditions and diseases. Cognitive impairment regarding the recognition of facial expressions of emotion is found in a variety of neurological and psychiatric disorders and is associated with a decline in social skills and quality of life. Accurate assessment and improvement of facial emotional recognition using a treatment focused on specific clinical symptoms, such as the misrecognition of emotions, can improve a patient’s quality of life.

However, the influences of gender, age, and culture on emotional recognition were obvious. Previous studies on facial expression recognition have shown that older people have reduced accuracy in recognizing negative emotions such as anger, sadness, and fear compared to younger people, while there were no consistent difficulties with happiness, surprise, or disgust [12, 13]. Westerners tend to accurately identify expressions of fear and disgust, which are among the six basic emotions considered universal, whereas East Asians cannot reliably identify them [14, 15]. The Japanese have been reported to tend to suppress emotional expressions more than Westerners [16, 17], and this suppression of emotional expressions has been reported to be stronger in men than in women [18]. When recognizing facial expressions, it has been shown that East Asians often look at the area around the eyes, while Westerners tend to look mainly at the area around the mouth [19, 20], suggesting that there may also be differences in the way facial expressions are recognized. These differences are thought to be rooted in differences in cultural and gender norms between countries and across generations, in addition to physical factors, such as aging. It has become clear that various factors influence facial expression recognition and that facial expression recognition varies by culture, gender, and generation. Thus, to assess emotion recognition in patients with affective disorders, it is necessary to investigate how these factors individually affect facial expression recognition before examining the characteristics of emotion recognition in diseases and symptoms.

Furthermore, the importance of recognizing moving images, in addition to the traditional use of facial expression recognition using still images, has recently been discussed. In the field of facial expression research, many studies have used still images of facial expressions. However, studies using dynamic images have recently begun [21–24]. It has been shown that facial expression recognition with dynamic images elicits stronger emotional responses than with still images [25, 26], and dynamic stimuli have been suggested to be useful in assessing psychiatric disorders that may impair emotion recognition [27, 28]. In recent years, a method for evaluating emotional recognition using the Facial Acting Coding System (FACS) has gained international recognition [29–31] and is expected to be utilized in the medical field [32, 33]. The FACS is a system for classifying human facial movements by appearance and is used as a standard for systematically classifying physical expressions of emotions. It is a computed, automated system that detects faces in videos, extracts facial geometric features, and creates a temporal profile of each facial movement. Humans recognize emotions through the observation of facial expressions in motion, and we believe that video-based indicators are more representative of the reality of facial expressions.

We investigated the emotional recognition of healthy young Japanese adults in response to videos of facial expressions. We chose 20–23-year-old young adult university students as the subjects because we expected them to have less variability in emotion recognition function than younger or elderly people [34–38]. Verification of Japanese characteristics in facial expression recognition, including the judgment of emotional categories and emotional intensities, is essential for indicating clinical treatments and therapeutic assessments for Japanese psychiatric patients. We investigated the characteristics of facial expression recognition in young Japanese individuals to establish a video-based facial expression recognition assessment method for Japanese populations. Because it has been reported that patients with psychiatric disorders, including affective disorders, perceive the intensity of emotion [39, 40] as well as the type of emotion [41, 42] differently from healthy controls, we evaluated both the intensity and type of emotion.

Although own-age and own-race biases have been found in facial expression recognition [43–46], the influence of the gender of the face as a stimulus has rarely been discussed. In the present study, we hypothesized that the gender of the stimuli might affect emotion recognition, as the age and race of the stimuli affect emotion recognition. We investigated the effects of the gender of the evaluator and the stimulus face on facial expression recognition.

## Methods

Healthy Japanese participants were asked to identify emotions and evaluate the intensity of emotions after viewing videos of facial expressions.

### Pre-experiment

Researchers at the University of Glasgow created models of 2,400 facial expressions by manipulating random facial muscles using FACS. Each model expression took the form of an animated image that changed from an emotionally neutral face to an emotional expression, and then back to a neutral face through manipulated changes in the facial muscles. In each model, the facial expression had a peak at approximately 0.5 s after the beginning of the video. The entire sequence took approximately 1.25 s. The evaluators in our study, 12 healthy Japanese undergraduates (6 males and 6 females; mean age 21.3 ± 0.6 years), responded to each of the six basic emotions (happiness, sadness, anger, fear, disgust, surprise) in each of the 2,400 models. We selected the top 17 models for each emotion judged by the evaluators as the same emotion and manipulated the models using FACS to achieve three levels of emotional intensity.

### Experiment

#### Participants

The participants in the main experiment were 30 healthy Japanese university students (15 males; mean age 21.4 ± 1.1 years, 15 females; mean age 21.1 ± 0.6 years) who had not participated in the pre-experiment. None of the participants had visual impairments, including loss of vision, autism spectrum disorders, prosopagnosia, or other characteristics that could cause difficulty in recognizing emotions. All participants were free of learning disabilities or synesthesia. All participants had been exposed to Japanese culture throughout their lives, and none had spent long periods outside Japan or had been exposed to non-Japanese culture for extended periods.

#### Materials

In total, 306 facial expressions (6 emotions × 17 expression models × 3 levels of intensity) set up in the pre-experiment were applied to the four Japanese faces (two for each gender), creating 1,224 animated images of facial expressions. A monitor (23.6"; aspect ratio 16:3; resolution 1920 × 1080 ppi) displaying images of facial expressions was placed 69 cm away from the participants, and the visual angle to the displayed faces was 14.4° vertically and 9.7° horizontally. Each display had a black background. The experiment was performed in a quiet, externally light-free, shielded room. Each participant sat in a chair with their chin placed on a desk chinrest and held a computer mouse with the dominant hand.

#### Procedure

**Fig. 1:**
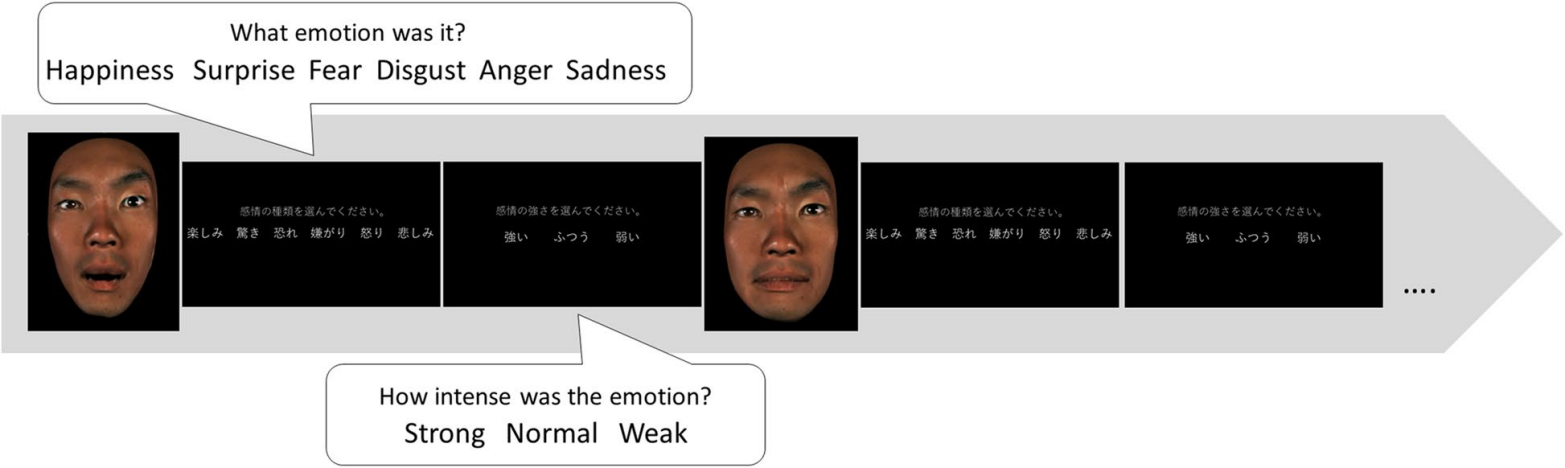
Experimental design. Participants judged the emotional type and evaluated the emotional intensity of each animated image

For the 1,224 facial expression videos, the participants judged the emotional type and evaluated the intensity of each animated image. The images were randomly divided into 51 × 4 blocks × 6 sessions and the participants took breaks between sessions when appropriate. Each image was presented only once. Options for the type of emotion (happiness, surprise, fear, disgust, anger, and sadness) and intensity (strong, intermediate, or weak) were displayed on the screen. After a choice was made, the next image was displayed (Fig. 1). The images were shown in a random sequence. We used PsychoPy3 (University of Nottingham) to present the images and obtain responses.

#### Statistical analysis

First, we calculated the mean and standard deviation of accuracy, that is, the percentage of cases in which the evaluators chose the same type of emotion as intended. Second, an ANOVA with three factors (the gender of facial images, the gender of evaluators, and emotion) was performed using SPSS (version 27) to reveal the gender effect of the evaluators and stimulus faces.

Third, the intensity of emotions was analyzed. An analysis was performed for each emotion type. We set the intensity of the emotion to 1 point for weak, 2 points for intermediate, and 3 points for strong. The value of the intensity evaluated by the evaluator was subtracted from the intended intensity and used for further analysis of the emotional intensity. The mean and standard deviation of the values were calculated, and an ANOVA was performed to reveal the gender effect of the evaluators and stimulus faces.

## Results

### Evaluation of the types of emotions

The accuracy with which each emotion was assessed was as follows: happiness, 97.9%; sadness, 54.2%; anger, 65.5%; fear, 19.4%; disgust, 68.2%; surprise, 91.8%. Except for fear, the evaluators were more than 60% accurate for approximately half of the videos. However, the highest level of accuracy for facial expressions of fear was only 57%. A few fear images were recognized as such.

**Fig. 2:**
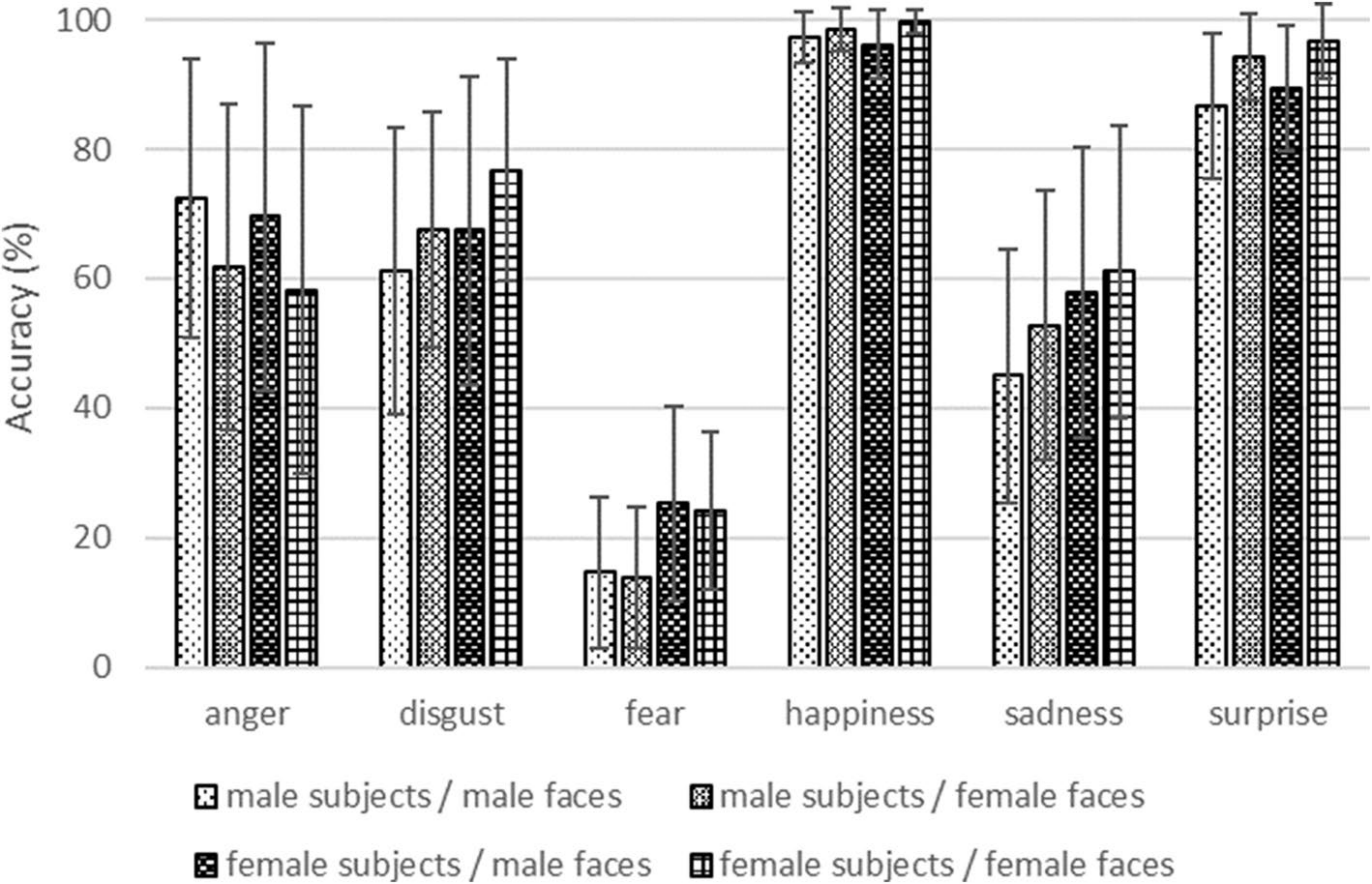
Accuracy depends on the gender of the faces and the evaluators. Female faces were more recognizable for most emotions, except anger and fear, while male faces were more recognizable when they showed anger. Female evaluators were able to identify most emotions accurately, except for happiness and anger. Notes: Error bars indicate standard errors

ANOVA showed the main effect of emotion (F (5, 2376) = 1016.47, *p* < 0.001, η2p = 0.681), the gender of facial images (F (1, 2376) = 6.87, *p* = 0.009, η2p = 0.003), and the gender of evaluators (F (1, 2376) = 44.07, *p* < 0.001, η2p = 0.018) (Table 1). Interactions were found between the gender of facial images and emotion (F (5, 2376) = 17.25, *p* < 0.001, η2p = 0.035), and between the gender of evaluators and emotion (F (5, 2376) = 11.10, *p* < 0.001, η2p = 0.023), but not between the gender of facial images and that of evaluators (F (1, 2376) = 0.003, *p* = 0.957, η2p = 0.000) (Table 1). The individual analysis for each emotion showed the main effects of the gender of facial images, except in the case of fear (happiness; F (1, 404) = 36.93, *p* < 0.001, η2p = 0.084: sadness; F (1, 404) = 6.83, *p* = 0.009, η2p = 0.017: anger; F (1, 404) = 18.72, *p* < 0.001, η2p = 0.044: fear; F (1, 356) = 0.57, *p* = 0.450, η2p = 0.002: disgust; F (1, 404) = 15.03, *p* < 0.001, η2p = 0.036: surprise; F (1, 404) = 75.91, *p* < 0.001, η2p = 0.158), and the main effects of evaluators’ gender except for faces with happy and angry expressions (happiness; F (1, 404) = 0.00,* p* = 1.000, η2p = 0.000: sadness; F (1, 404) = 25.01, *p* < 0.001, η2p = 0.058: anger; F (1, 404) = 1.63, *p* = 0.203, η2p = 0.004: fear; F (1, 356) = 63.38, *p* < 0.001, η2p = 0.151: disgust; F (1, 404) = 14.49, *p* < 0.001, η2p = 0.035: surprise; F (1, 404) = 9.28, *p* = 0.002, η2p = 0.022) (Table 2). These results indicate that female faces were more recognizable when they showed happiness, sadness, disgust, and surprise, whereas male faces were more recognizable when they showed anger. Female evaluators accurately recognized sad, fearful, disgusted, and surprised faces (Fig. 2). An interaction was observed between the gender of the faces and the evaluators of happy faces (F (1, 404) = 9.77, *p* = 0.002, η2p = 0.024), suggesting that female evaluators had the highest accuracy for happy female faces and the lowest accuracy for happy male faces.

**Table 1:** The ANOVA analysis for the gender of facial images, gender of evaluators, and emotion

		F value	*p* value	η^2^p
Main effect	gender of facial images; F(1, 2376)	6.87	0.009*	0.003
	gender of evaluators; F(1, 2376)	44.07	< 0.001*	0.018
	emotion; F(5, 2376)	1016.47	< 0.001*	0.681
Interaction	gender of facial images × gender of evaluators; F(1, 2376)	0.003	0.957	0.000
	gender of facial images × emotion; F(5, 2376)	17.25	< 0.001*	0.035
	gender of evaluators × emotion; F(5, 2376)	11.10	< 0.001*	0.023
	gender of facial images × gender of evaluators × emotion; F(5, 2376)	0.63	0.678	0.001

^*^p < 0.05

**Table 2:** The individual ANOVA analysis for each emotion

			F value	*p* value	η^2^p
Type of emotion	gender of facial images	anger; F (1, 404)	18.72	< 0.001*	0.044
		disgust; F (1, 404)	15.03	< 0.001*	0.036
		fear; F (1, 356)	0.57	0.450	0.002
		happiness; F (1, 404)	36.93	< 0.001*	0.084
		sadness; F (1, 404)	6.83	0.009*	0.017
		surprise; F (1, 404)	75.91	< 0.001*	0.158
	gender of evaluators	anger; F (1, 404)	1.63	0.203	0.004
		disgust; F (1, 404)	14.49	< 0.001*	0.035
		fear; F (1, 356)	63.38	< 0.001*	0.151
		happiness; F (1, 404)	0.00	1.000	0.000
		sadness; F (1, 404)	25.01	< 0.001*	0.058
		surprise; F (1, 404)	9.28	0.002*	0.022
Intensity of emotion	gender of facial images	anger; F (1,4001)	3.22	0.073	0.001
		disgust; F (1,4163)	1.36	0.243	0.000
		fear; F (1,1045)	6.27	0.012*	0.006
		happiness; F (1,5984)	130.78	< 0.001*	0.021
		sadness; F (1,3308)	1.77	0.184	0.001
		surprise; (F (1,5603)	26.45	< 0.001*	0.005
	gender of evaluators	anger; F (1,4001)	5.55	0.020	0.001
		disgust; F (1,4163)	84.74	< 0.001*	0.020
		fear; F (1,1045)	0.09	0.770	0.000
		happiness; F (1,5984)	22.45	< 0.001*	0.004
		sadness; F (1,3308)	4.32	0.038*	0.001
		surprise; F (1,5603)	25.68	< 0.001*	0.005

^*^p < 0.05

### Evaluation of the intensity of emotion

**Fig. 3:**
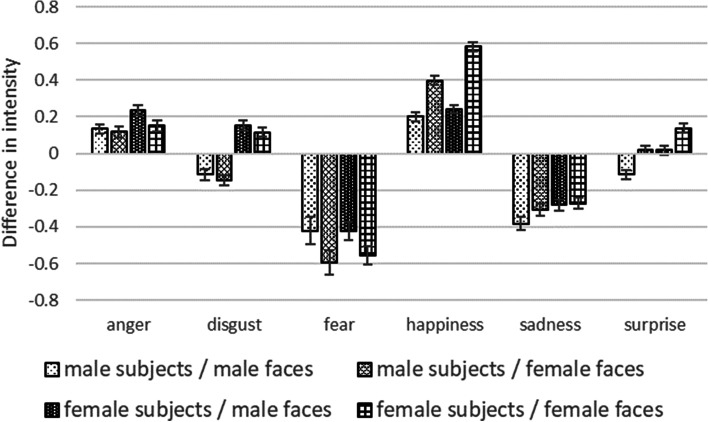
Gender differences in recognition of emotional intensity. The intensity of the emotion was set to 1 point for weak, 2 points for intermediate, and 3 points for strong. The differences in intensity were calculated by subtracting the intended value from the evaluated value. The expression of fear was judged to be less intense. Female evaluators tended to perceive most emotions as being more intense than male evaluators. Notes: Error bars indicate standard errors

We analyzed the evaluators’ judgments of the intensity of the emotion in the images only in cases where they accurately judged the type of emotion. We set the intensity of the emotion to 1 point for weak, 2 points for intermediate, and 3 points for strong. An ANOVA was used to analyze the difference between the intended score in the video and the evaluator’s score. The main effects of the face’s gender on the evaluation were observed for happy (F (1,5984) = 130.78, *p* < 0.001, η2p = 0.021), fearful (F (1,1045) = 6.27, *p* = 0.012, η2p = 0.006), and surprised (F (1,5603) = 26.45, *p* < 0.001, η2p = 0.005) faces, while sad (F (1,3308) = 1.77, *p* = 0.184, η2p = 0.001), angry (F (1,4001) = 3.22, *p* = 0.073, η2p = 0.001), and disgusted (F (1,4163) = 1.36, *p* = 0.243, η2p = 0.000) faces did not show it (Table 2). The main effect of the gender of the evaluators was observed for happy (F (1,5984) = 22.45, *p* < 0.001, η2p = 0.004), sad (F (1,3308) = 4.32, *p* = 0.038, η2p = 0.001), disgusted (F (1,4163) = 84.74, *p* < 0.001, η2p = 0.020), and surprised (F (1,5603) = 25.68, *p* < 0.001, η2p = 0.005) faces, while angry(F (1,4001) = 5.55, *p* = 0.02, η2p = 0.001) and fearful(F (1,1045) = 0.086, *p* = 0.77, η2p = 0.000) faces did not show it (Table 2). These results suggest that even though the movements of male and female facial expressions were the same, female faces were recognized as being more intense in terms of happy and surprised expressions. They were recognized as less intense in their expression of fear. Female evaluators perceived happy, sad, disgusted, and surprised expressions to be more intense than the male evaluators (Fig. 3). An interaction between the gender of the face and that of the evaluators was observed for happy faces, which indicated the highest intensity when female evaluators evaluated female faces and the lowest intensity when male evaluators evaluated male faces.

## Discussion

We focused on the factors that influence the evaluation of emotional facial expressions by healthy Japanese subjects to develop a method for assessing the pathophysiology of psychiatric disorders. In particular, we investigated the influence of the gender of the faces and evaluators on emotional recognition.

The results showed that females had a superior ability to recognize facial emotions than males. The gender of the faces influenced the evaluators’ judgment, with male faces being more recognizable for anger and female faces being more recognizable for other emotions. The evaluation of emotional intensities also differed according to the gender of the faces and evaluators.

### Evaluators’ gender

Our results showed that females were more accurate than males in recognizing emotions from facial expressions, especially sadness, fear, disgust, and surprise. This is consistent with previous studies suggesting females’ higher accuracy in emotional recognition [47–49]. Females are generally considered more social than males [50–52], and our results may reflect this finding.

People recognized happiness more easily when the gender of the face and evaluators matched. People tend to have their own gender biases when recognizing other faces. In particular, females tend to be better able to recognize female faces more accurately than male faces [53]. The own-gender bias of male evaluators has not yet been clarified [53]; however, for obvious facial expressions such as happiness, this bias could be enhanced for males as well, resulting in higher accuracy recognition of facial expressions for the same gender.

### Facial gender

The facial expression videos for this study were created by applying a specific facial expression model to both male and female faces, eliminating any differences in the facial movements in the expression of the male and female faces. However, we identified a gender difference in the accuracy of the evaluations, suggesting that facial gender affects the accuracy of expressed emotions. Female faces were more recognizable in happy, sad, disgusted, and surprised expressions, whereas male faces were more recognizable in angry expressions. To interpret this sex difference, we developed the following two hypotheses. First, female evaluators, who are considered to have superior emotion recognition abilities, tend to recognize female faces more easily because of gender bias. Females have a higher degree of sociality in recognizing emotions than males. Own-sex advantages and higher sociality may contribute to greater accuracy when observing female faces. Another reason for this is the influence of Japanese culture, which discourages the overt expression of emotions. A previous study [54] found that American students expressed disgust when viewing highly stressful films, whereas Japanese students concealed their feelings. It has also been suggested that this suppression of emotional expression in Japanese students is stronger in males than in females [55]; females express their feelings with their facial expressions more than males do, which may imply that the Japanese may have greater familiarity with perceiving emotions on female faces than on male faces. The evaluators may have found it easier to recognize facial expressions in female faces. Likewise, males tend to express their anger more than females [55], making angry faces more recognizable. In this case, the accuracy with male angry faces was greater than for female faces. Previous studies have shown that the expression of happiness is recognized more accurately and quickly on female faces than on male faces and that male faces expressing anger are recognized more accurately and quickly than female faces expressing anger [56, 57]. Our results are consistent with these findings. Likewise, the present study found that the sex of the face made a difference in recognizing emotions, even when there were no differences in facial muscle movements.

### Emotional intensity

The female evaluators judged the intensity of the emotions to be stronger than the male evaluators regarding the expressions of happiness, sadness, disgust, and surprise. A previous study on Chinese adolescents reported that female participants judged emotionally negative and positive photos to have a higher intensity than male participants did [58]. However, this finding varies by age and culture. A German study reported that females perceive emotionally negative photos to be more intense than males, but emotionally positive photos are less intense [58]. The difference in the gender of the evaluators when recognizing emotional intensity is not universal and varies according to gender norms and stereotypes based on culture and generation. For the Japanese evaluators in the present study, females judged the intensity of the faces expressing happy, sad, disgusted, and surprised emotions to be stronger than males, consistent with the Chinese study. This might be related to the fact that the culture, including gender roles in Japan, is similar to that of China [59].

The evaluators recognized female faces as having more intensity for happiness and surprise, whereas male faces were recognized as more intense for fear. Both male and female faces were presented with the same facial muscle movements, and this unexpected result regarding the intensity of emotions between male and female faces may reflect the evaluators’ gender preferences. Gender stereotypes [60, 61], such as “Females are emotional” and “Males should not be vulnerable,” may have influenced the evaluators more in the context of the display of more potent emotions such as happiness and surprise, resulting in a difference in the evaluators’ recognition of the emotional intensity.

Another factor that potentially influenced the evaluation of emotional intensity was related to the different features of the male and female faces. Although the relationship between facial features and emotion recognition remains unclear, reports have shown that masculine features, such as a square jaw, thick eyebrows, and a high forehead, match features expressing anger [56, 62]. The construction features of the male and female faces may have influenced the evaluators’ emotional intensity results. A previous study using functional magnetic resonance imaging found that when a male observes a female face, the activity of the amygdala increases independent of the type of emotion expressed in the image. However, the reverse does not hold when a woman is looking at a male face or when any person, regardless of the observer’s sex, is looking at a face of the same gender, [63]. This difference in brain activity may result in differences in the perception of emotional intensity and the emotions themselves.

### Differences by type of emotion

The accuracy with which each emotion was assessed was as follows: happiness, 97.9%; sadness, 54.2%; anger, 65.5%; fear, 19.4%; disgust, 68.2%; surprise, 91.8%. The evaluators identified only 19.4% of the facial expressions that expressed fear. In a few cases, the evaluators judged other facial expressions as fear. Fear was identified in 6.18% of all facial expression images, well below the expected percentage of 16.7%. These results are consistent with previous studies showing that Japanese people are less likely to recognize fear [64] and less accurate in judging fear as fear [64, 65], suggesting that fear is less recognizable by the Japanese. However, 46% of the facial expression images that were judged as fearful were designed to have the fearful expression. This indicates that Japanese people can recognize facial expressions of fear as fear but are less likely to do so compared to other emotions. Fear tended to be confused with sadness and disgust.

**Fig. 4:**
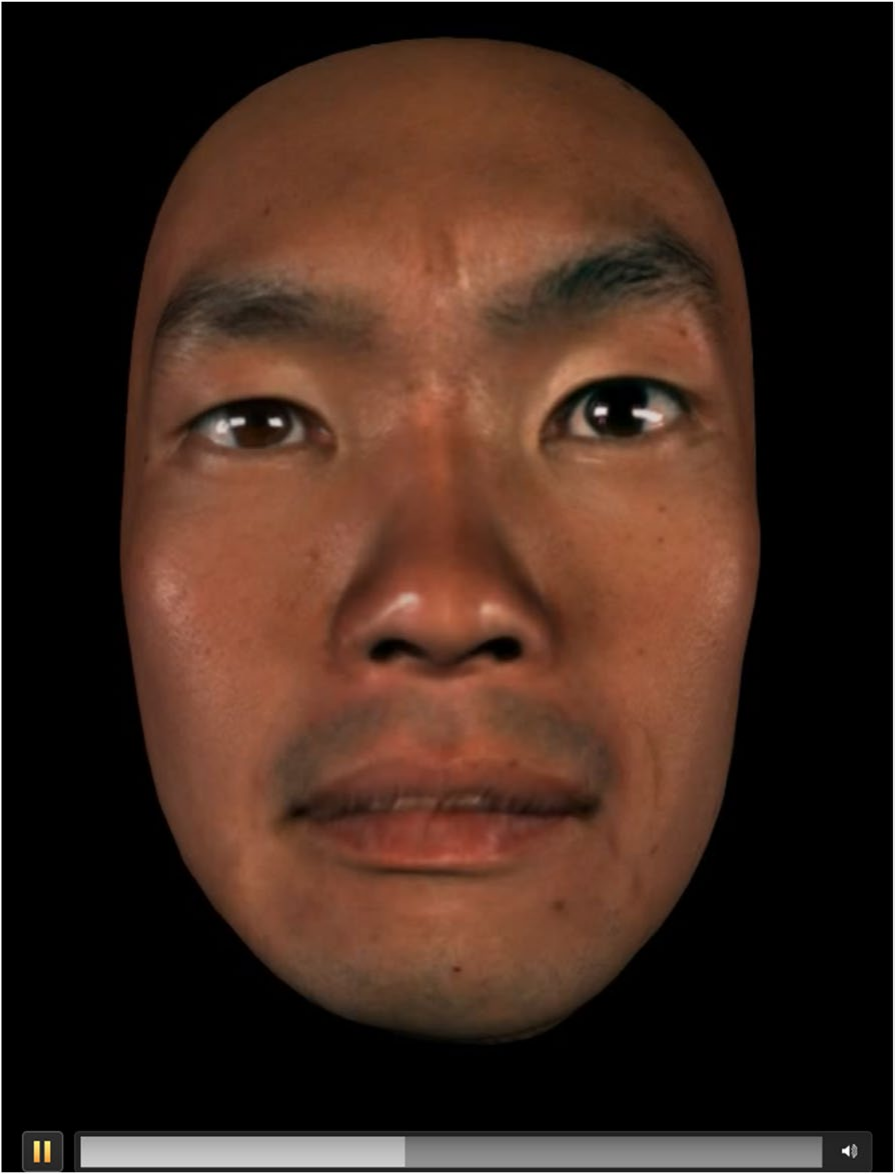
The facial expression evaluated as fear with the highest accuracy. This facial expression includes eyebrow and lip features that characterize the expression of fear, but it is not a typical facial expression of fear in other respects

According to Paul Ekman [66], the facial expression of fear is likely to be confused with surprise, as its characteristics are as follows: 1) eyebrows raised and pulled together, 2) raised upper eyelids, 3) tense lower eyelids, and 4) open and dropped jaws and lips stretched horizontally backwards. The most “fear-like” image (Fig. 4) in this study included these eyebrow and lip features, but it was not a typical facial expression of fear in other respects. However, the facial expressions identified as fear, including those representing other emotions, shared the same eyebrow action. Previous studies showed that Westerners pay closer attention to mouth movements when recognizing emotions from facial expressions, while most Japanese pay closer attention to eye movements [20, 67]. Consistent with these findings, our results suggest that Japanese people tend to infer emotions mainly from the movements of the eyebrows and eyes rather than from the movements of the mouth and jaw.

### Clinical application of facial recognition assessment and future directions of the study

This study aimed to establish a method for assessing psychiatric disorders for Japanese populations using facial expression recognition. However, because various factors such as cultural and gender differences are involved in facial expression recognition, it is necessary to investigate the details of these factors to establish an assessment scale. In this preliminary study, we investigated sex differences in facial expression recognition in a single age group exposed to a single culture. Although studies assessing facial expression recognition in psychiatric disorders usually control for age and race, only a few have discussed gender bias. In this regard, we believe it is significant that this study clarified that not only the gender of the evaluator but also that of the facial stimuli affected the evaluation of facial expressions.

In addition, the young adult participants in this study were culturally and age homogeneous. Young adults are theoretically at the peak of cognitive functioning, and future similar experiments with subjects of different age groups could provide a basis for investigating physiological and pathological age-related changes in facial emotion recognition. The results of this study shed light on age-related deficits in emotion recognition and additional impairments underlying pathophysiological neural processes. Neurophysiological activity in facial emotion recognition has been reported to vary with the evaluator’s age [68, 69], sex [70], and even culture [71]. Our future work, using a cultural- and age-unified dataset and neurophysiological techniques, may enable the identification of core neural networks associated with emotion recognition.

### Limitations of this study

This study has some limitations. First, the sample size is small. A small sample size leads to high variability, which may lead to bias. The number of subjects must be increased for a discussion with certainty. Second, the participants in this study were young. It has been suggested that own-age bias is involved in facial expression recognition [43, 44], and if an assessment of facial expression recognition is used as a scale to evaluate mental status, it is necessary to confirm how facial expressions are recognized, not only in younger people but also in elderly people. The third category includes facial expressions that express fear. Unfortunately, the evaluators judged facial expressions intended as fearful incorrectly. Japanese people are indeed able to recognize facial expressions of fear but are less likely to do so compared to other emotions. Further studies should be conducted to investigate the facial expressions that Japanese people recognize as fearful.

## Conclusions

This study investigated factors influencing the emotional evaluation of facial moves. Although the gender of the evaluators and the gender of the faces influenced how the evaluators judged emotions, the emotional facial movies used in this study, except for fear, were evaluated accurately, suggesting that these videos could be used as a dataset. This study has some limitations regarding sample size and specific samples. In contrast, it did show that females tended to recognize the intended emotion more accurately and intensely than males, at least for young, healthy Japanese evaluators. Female faces were more recognizable than male faces. As these inclinations might be related to gender norms, age, and culture, further studies on other age groups and cultures are required to create a more comprehensive dataset. In particular, fear was difficult for the participants, who were all Japanese, to recognize. Thus, it is necessary to consider the facial features that a Japanese participant group would recognize as expressing fear.

## Data Availability

The datasets used and analyzed in the current study are available from the corresponding author upon reasonable request.

## Abbreviations

FACS: Facial Acting Coding System
ANOVA: Analysis of variance
